# Evidence of the Giant Barocaloric Effect in the PVA-Slime System by Molecular Dynamics Simulations

**DOI:** 10.1021/acsomega.5c02475

**Published:** 2025-08-23

**Authors:** Richard Javier Caraballo-Vivas, Marcelo Albuquerque, Vanessa Torres, Luciano T. Costa, Pedro Venezuela, Mario Reis

**Affiliations:** † Institute of Physics, 28110 Universidade Federal Fluminense, Av. Gal. Milton Tavares de Souza s/n, Niterói-RJ 24210-346, Brazil; ‡ MolMod-CS-Department of Chemistry, Universidade Federal Fluminense, Outeiro de São João Batista, 24020-141 Niterói RJ, Brazil

## Abstract

Advancements in the study of the barocaloric effect in polymers have opened promising applications in both the scientific and industrial fields. Among these, elastic polymers based on poly­(vinyl alcohol) (PVA), such as slimes, have shown significant potential for solid-state refrigeration and thermal battery applications due to their notable pressure-induced thermal response, which occurs without an associated structural phase transition. Thus, current research focuses on understanding the mechanism behind this response to applied pressure with the aim of optimizing its thermal performance. Therefore, we employed a molecular dynamics simulation in order to explore the barocaloric effect in the Slime system. We used pure PVA chains cross-linked by tetrahydroxyborate ions to provide further details about our Slime system, promoting a greater proximity between polymeric chains. Our results reveal that these connections reduce the free volume in the Slime system compared to pure PVA. This, combined with the applied simulated pressure, decreases the mobility of the polymer chains, lowering their kinetic energy while favoring potential energy. As a result, this contributes significantly to the change in internal energy and, consequently, to the barocaloric effect. Thus, our investigation shows a significant increase in entropy from 56 JK^–1^ kg^–1^ for pure PVA to 295 JK^–1^ kg^–1^ Slime system and temperature change from 3 to 26 K at 300 MPa. These findings highlight the importance of cross-linking between polymer chains, which enhances the barocaloric effect in this system type, offering promising prospects for practical applications.

## Graphical abstract

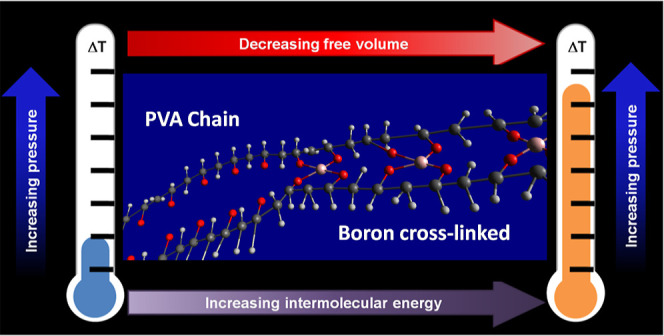

## Introduction

Poly­(vinyl alcohol) (PVA) is a synthetic polymer with a wide range of scientific, engineering, and industrial uses.^1–3^ Some of the most notable properties of PVA include its high solubility in water, mechanical resistance, biocompatibility, biodegradability, cost-effectiveness, and scalability.^4^ This versatility has been fundamental in the development of applications in diverse areas, such as biomaterials,^5–7^ manufacturing of nanocomposite devices,^8^ and green technologies,^9^ just to name a few. Cross-linking PVA chains can modify their intrinsic properties, such as water absorption capacity, chemical resistance, and mechanical strength, thereby enhancing their flexibility and imparting viscoelastic characteristics.^10^ This enhancement is achieved through the chemical addition of borax, which facilitates the formation of Slime and results in an optimal material for a range of soft matter applications, rendering it more effective for those who require both elasticity and strength such as flexible robots,^11^ decontamination environments,^12^ and electrolyte systems.^13^ Thus, the formation of Slime through the combination of PVA and borax not only enhances the mechanical properties of materials but also affects its thermal responses due to increasing cross-link density of chain interactions.^14^ Therefore, when external pressure is applied, this interaction becomes particularly relevant for the barocaloric effect and related technologies.

The modification of the mechanical properties of PVA through the cross-linking of its chains, particularly by adding borax to form a Slime system, is fundamental to exploring the barocaloric effect. This thermodynamic phenomenon involves the application of pressure to a material, which induces a change in its temperature or heat exchange with the environment.^15^ This cross-linking not only enhances the mechanical and viscoelastic characteristics of the material but also influences its thermodynamic behavior, even in the absence of an associated phase transition, as demonstrated by Bom and co-workers with natural rubber.^16^ Although no experimental data are yet available for the barocaloric effect in cross-linked PVA under pressure, similar materials like polydimethylsiloxane (PDMS),^17^ a highly flexible elastomeric polymer, have demonstrated a significantly greater barocaloric response under pressure.^18^ Considering that PVA shares similar mechanical and thermodynamic properties with PDMS, especially when borax is used as a cross-linker, it is reasonable to expect that PVA could exhibit comparable behavior and show considerable potential for barocaloric applications. By increasing the cross-link density, the interaction between polymer chains is strengthened, altering the internal energy and resulting in applications such as thermal batteries^19^ and cooling systems.^20,21^ Thus, investigating the relationship between polymer cross-linking and the barocaloric effect is crucial for developing promising materials with elastic properties, such as the PVA-Slime system.

Therefore, this work is a theoretical investigation of the barocaloric effect in a polymeric system based on poly­(vinyl alcohol), which has the polymeric chains cross-linked with tetrahydroxyborate ions, forming a Slime system. For this purpose, we employed all-atoms molecular dynamics simulations, which is a powerful tool to facilitate the prediction of the behavior of barocaloric materials,^22,23^ accelerating the development of compounds with optimized properties for applications in solid-state refrigeration and thermal energy storage. From this study, we examined how cross-linking affects the physical and thermodynamic properties of the system. We found that this cross-linking alters the mobility of the polymer chains, thereby influencing the intermolecular interaction of polymer chains under pressure.

For barocaloric applications, our findings include a significant enhancement in the entropy change and a linear relationship between the temperature change and applied pressure, demonstrating the effectiveness of tetrahydroxyborate addition in forming a Slime system. These insights contribute to the development of advanced materials for thermal management and highlight the potential of the Slime system in innovative cooling and heating technologies. Thus, this work is structured as follows: the Methods section describes the performed simulations, followed by an analysis in the Results and Discussion section of how cross-linked chains influence the thermodynamic properties of pure PVA and the Slime system along with the investigation of the barocaloric effect in polymer systems. Finally, the last section presents the concluding remarks.

## Methods

### Simulated Systems

The built polymer models were based on cross-linked poly­(vinyl alcohol) (PVA: ${\left[{CH}_{2}CH\left(OH\right)\right]}_{n}$ ) chains, which consist of PVA oligomers, forming a chain with 20 monomers, with methyl groups at both chain endings, resulting in a total molecular weight of approximately 897 g/mol. PVA has polymeric matrix properties that allow the preparation of chemically interlinked chains through tetrahydroxyborate ions [B­(OH)_4_^‑^], which introduce negative charges that aid intermolecular bonding between chains. Experimentally, B­(OH)_4_^‑^ ions are added through the chemical reaction of PVA with borax (Na_2_B_4_O_7_), which also introduces Na^+^ ions to balance the electrostatic charge of the solution. Based on these experimental facts, we successfully modeled the Slime system by chemical cross-linking of two polymer chains connected by B­(OH)_4_^‑^ ions, which showed fixed connectivity in order to reflect the gel-like state of the Slime system and prevent large-scale rearrangements of the polymer chains and were electrostatically balanced by Na^+^ ions with a molecular weight of approximately 870 g/mol, similar to the diagram shown in Figure 1a. These chains were used to generate initial cubic simulation boxes for pure PVA (without borax) and Slime systems, ensuring a tolerance limit of 2.0 Å as set by PACKMOL during the packing process.^24,25^ This resulted in initial simulation boxes containing 4350 atoms forming 30 chains and occupying 39 nm^3^ for the pure PVA system and 5850 atoms forming 50 chains and occupying 72 nm^3^ for the Slime system.

**Figure 1:**
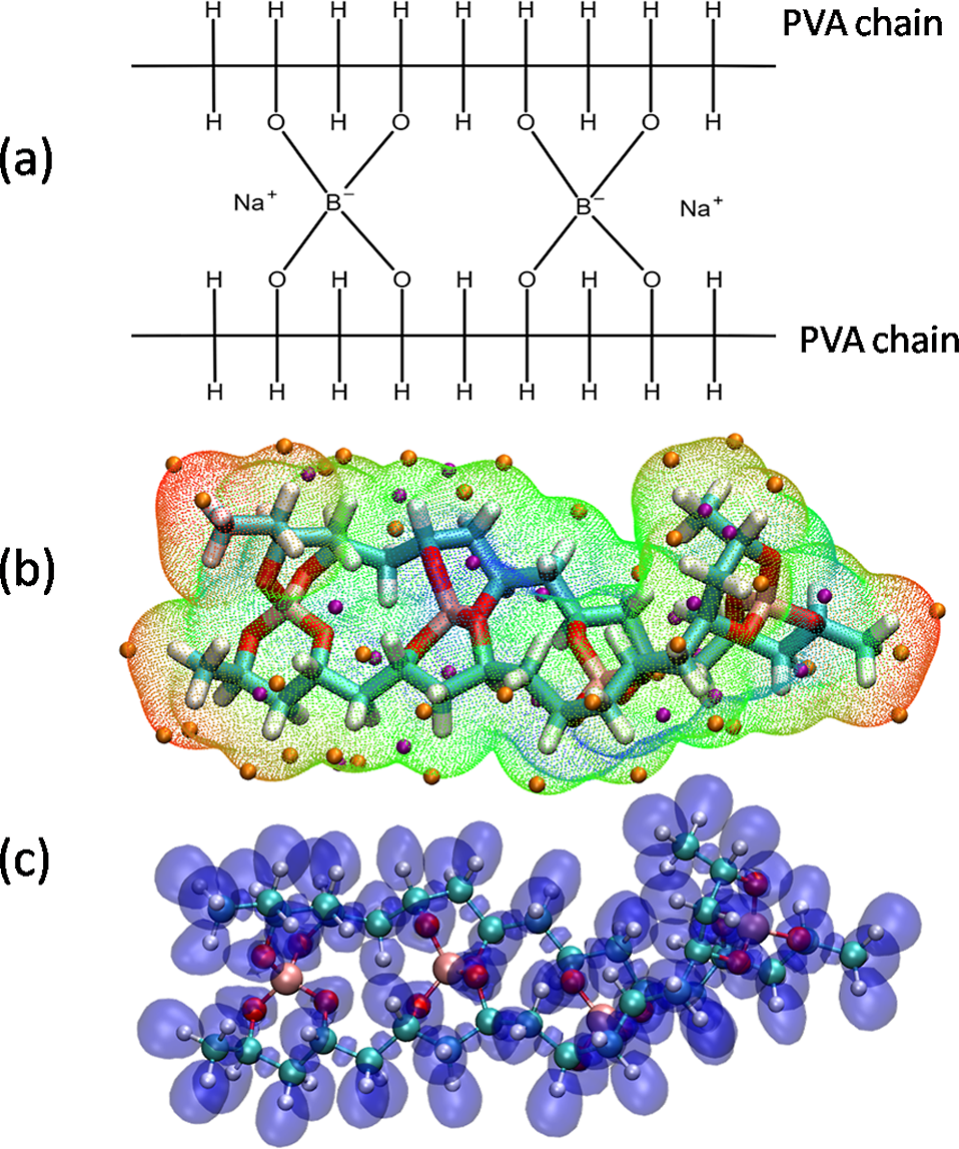
(Color online) Scheme of elaboration of the Slime system from pure PVA chains cross-linking with boron atoms (a). Electrostatic Potential Surface, EPS, calculated (b) and Electron Localization Function, ELF (c).

### Molecular Dynamics Simulations

Molecular dynamics simulations using GROMACS 2023.3 software^26^ were employed to solve Newton’s equations of classical motion for atomic constituents within the simulation boxes. Periodic boundary conditions (PBC) were applied in all dimensions, and the Verlet cutoff scheme was used with the default buffer size for neighbor searching.^27^ The systems modeled in this work were described using the Generalized AMBER Force Field (GAFF)^28^ to characterize intra- and intermolecular interactions of the systems’ constituents. Atomic point charges were derived from molecular density functional theory (DFT) calculations performed at the B3LYP/6–311++G** level of theory,^29–31^ which was realized on the anionic Slime system with a net charge of −4, excluding Na cations within implicit solvation using the Conductor-like Polarizable Continuum Model (CPCM)^32^ during both geometry optimization and charge derivation. Optimization followed by frequency calculation was performed using the software ORCA version 5.0.3.^33,34^ No imaginary values were found after the optimization, and single-point calculations were performed to obtain the CHELPG atomic charges,^35^ which were used to compose the atomic charges in the molecular dynamics simulations. The postprocessing software Multiwfn^36^ was employed to calculate the electrostatic potential surface (ESP),^37,38^ as shown in Figure 1b. Thus, we obtain the critical points at which it is possible to spot the low and high values of the potential represented by the purple and orange points, respectively. In addition, Multiwfn was used to calculate the electron localization function (ELF),^39^ which visually highlights regions where electron motion is most likely to occur, as shown in Figure 1c. Combining this result with the surface of the electrostatic potential (ESP) indicates that the regions where cation interactions are expected are near the B­(OH)_4_^‑^ groups. An isovalue of 0.75 e/Å^3^ was adopted, and the visual molecular dynamics (VMD)^40^ software was used to visualize the molecular configuration, ELF, and ESP surfaces.

To equilibrate the investigated systems, the following protocol was implemented.

(i) Energy minimization using the steepest descent algorithm, with the minimization stopped when the maximum force is less than 40 kJ mol^–1^ nm^–1^.
(ii) Simulated annealing in the canonical ensemble (NVT),^41^ employing a V-rescale thermostat with canonical sampling^42^ with a coupling time constant of τ_*t*_ = 0.1 ps. The protocol consists of equilibration at 300 K for 1 ns, heating from 300 to 800 K over 5 ns, maintaining 800 K for 1 ns, cooling back to 300 K over 5 ns, and a final equilibration at 300 K for 1 ns.
(iii) Conjugate gradient algorithm for energy minimization.
(iv) An equilibrium NpT ensemble run with a C-rescale barostat,^43^ maintaining isobaric conditions at ambient pressure with a coupling constant of τ_*p*_ = 1 bar and isothermal conditions at 300 K for 5 ns, was conducted to release any possible tension. These last simulations concerning the equilibrated systems are shown in Figure S1 in the Supporting Information (S1).

Then, production run simulations were performed using a V-rescale thermostat with canonical sampling^42^ over the temperature range from 200 to 500 K, with a step of 25 K, where a separate NpT simulation was conducted at each temperature, and a C-rescale barostat^43^ from room pressure to 500 MPa, with pressure steep of 100 MPa. Thus, thermodynamics parameters were determined by NpT ensemble simulations with a standard duration of at least 2 ns and statistically calculated over the last 1 ns interval. The simulation parameters and topology files are provided in the Supporting Information (S1) for a better understanding and reproducibility of the results.

### Estimation of Parameters

From the production process, we conducted thermodynamic and structural analysis using the GROMACS tools.^26,44–46^ The key quantity for barocaloric effect calculations was the volume box change Δ*V*, while variations in temperature *T*, and pressure *p*, parameters were also applied. The Radial Distribution Function (RDF) analysis was conducted to investigate the effect of cross-linking density on the local structure. This consists of probing how the local structure of the simulated systems behaves in terms of intra- and intermolecular packing toward cross-linked complexes. RDFs, $g_{A,B}\left(r\right)$ , are defined as the probability of finding a given particle B at the vicinity (within a radius *r*) of a reference particle A , defined as^47^

1 $$g_{A,B}\left(r\right)=\frac{1}{\rho_{B}}\frac{1}{N_{A}}\sum_{i,j}^{N_{A},N_{B}}\frac{1}{4\pi r^{2}}\delta \left(r_{ij}-r_{0}\right)$$

where *i* and *j* refer to the *i*th and *j*th atoms of the group A of $N_{A}$ atoms and B of $N_{B}$ atoms, respectively, while $\rho_{B}$ is the average density in all angle slices from θ to θ + dθ up to *r*_max_.

From the analysis of the trajectories during the simulation, we determined the mean square displacement, MSD, which investigates the dynamics of the systems by evaluating the average squared displacement of particles as a function of time, so that

2 $$MSD\left(\tau \right)=\langle \Delta {\mathrm{r⃗}}_{i}{\left(\tau \right)\rangle }^{2}=\langle {\mathrm{r⃗}}_{i}\left(\tau \right)-{\mathrm{r⃗}}_{i}{\left(0\right)\rangle }^{2}$$

where ${\mathrm{r⃗}}_{i}\left(\tau \right)$ and ${\mathrm{r⃗}}_{i}\left(0\right)$ represent the position of particle *i* after a time τ and the initial time, respectively. This analysis provides insights into molecular mobility and diffusive behavior, which are inherently tied to the available free volume in the simulation box, as a larger free volume allows greater molecular displacement and higher diffusion.

Physical quantities related to volume variations due to applied pressure to the barocaloric effect are the entropy change, Δ*S*, and the temperature adiabatic change, Δ*T*. Generally, these are found by the Clausius–Clapeyron relation for phase transformation.^23,48,49^ However, through molecular dynamics simulation, we are able to monitor both volume change (Δ*V*) and internal energy (*U*), variation systems under simulated applied pressure (*p*).^22^ Thus, we can evaluate directly the rate of change of entropy and temperature for pressure from the first law of thermodynamics *dU* = TdS–*pdV*

3 $${\left(\frac{\partial S}{\partial p}\right)}_{T}=\frac{1}{T}{\left(\frac{\partial U}{\partial p}\right)}_{T}+\frac{p}{T}{\left(\frac{\partial V}{\partial p}\right)}_{T}$$

4 $${\left(\frac{\partial T}{\partial p}\right)}_{S}=-\frac{1}{C_{p}}{\left(\frac{\partial U}{\partial p}\right)}_{S}-\frac{p}{C_{p}}{\left(\frac{\partial V}{\partial p}\right)}_{S}$$

where *C*_*p*_ is the constant pressure heat capacity, which can be obtained from the fluctuations in MD simulation.^50^ Similarly, it is possible to find other fluctuating quantities such as the thermal expansion coefficient α, isothermal compressibility κ_*T*_, and adiabatic bulk modulus *K*.^51^

Therefore, the isothermal entropy change and adiabatic temperature change upon compression from initial pressure *p*_0_, up to a final pressure applied *p*_*f*_, is obtained by the integration

5 $$\Delta S=\int_{p_{0}}^{p_{f}}\left[\frac{1}{T}{\left(\frac{\partial U}{\partial p}\right)}_{T}+\frac{p}{T}{\left(\frac{\partial V}{\partial p}\right)}_{T}\right]dp$$

6 $$\Delta T=\int_{p_{0}}^{p_{f}}-\left[\frac{1}{C_{p}}{\left(\frac{\partial U}{\partial p}\right)}_{S}+\frac{p}{C_{p}}{\left(\frac{\partial V}{\partial p}\right)}_{S}\right]dp$$

thus exploring the barocaloric effect.

## Results and Discussion

### Impact of Cross-Linked Chains on the Thermodynamic Properties of Polymers

To begin our analysis, we will first consider the volume variation behavior with temperature of the pure PVA and Slime systems and then how this is affected by the simulated pressure. To accomplish this, volume change will be the crucial parameter for the barocaloric effect investigation of compounds as it directly influences the thermal response of polymeric materials under pressure and temperature changes. Typically, the relationship between volume and temperature in polymers presents three distinctive regions: glassy, rubbery, and melting.^52^ The glassy region is characterized by a structurally rigid and fragile state due to limited molecular mobility. When the temperature approaches the glass transition temperature (*T*_g_), the material exhibits characteristics of the viscoelastic region in which the polymer gradually softens, transitioning from a hard, glass-like behavior to a more flexible, rubber-like state. Thus, we define the viscoelastic region as a transitional range in which the system retains glassy characteristics but exhibits an increasing molecular mobility as it approaches *T*_g_. This intermediate state combines the features of both glassy and rubbery behavior, enabling phenomena such as shape memory, where polymers recover their original shape after deformation.^53^ Their behavior can vary with temperature and the rate of applied stress or pressure, which is extremely important for barocaloric materials.^54^

Figure 2 illustrates the relative volume change, Δ*V*/*V*_0_, as a function of simulated temperature for both PVA and Slime at room pressure (≈0.1 MPa). In these systems, at least three distinct regions can be observed where the slope of the curve changes. These changes can be associated with region transitions due to temperature variations, identifiable as the glass region, viscoelastic region, and rubbery region, similar to what was proposed by Aklonis for amorphous polymers.^54^ The glass region for pure PVA and Slime systems extends from 200 K (the lowest simulated temperature) up to 250 and 275 K, respectively, and from these temperatures up to the glass transition temperature, *T*_g_, corresponding to the viscoelastic region. Above *T*_g_, the rubbery region begins, where the glass transition temperature for pure PVA has been experimentally observed to range between 320 and 375 K.^55–57^ We determined *T*_g_ for pure PVA to be 350 K, which aligns with the value calculated by molecular dynamics simulation by Noorjahan and Choi.^58^

**Figure 2:**
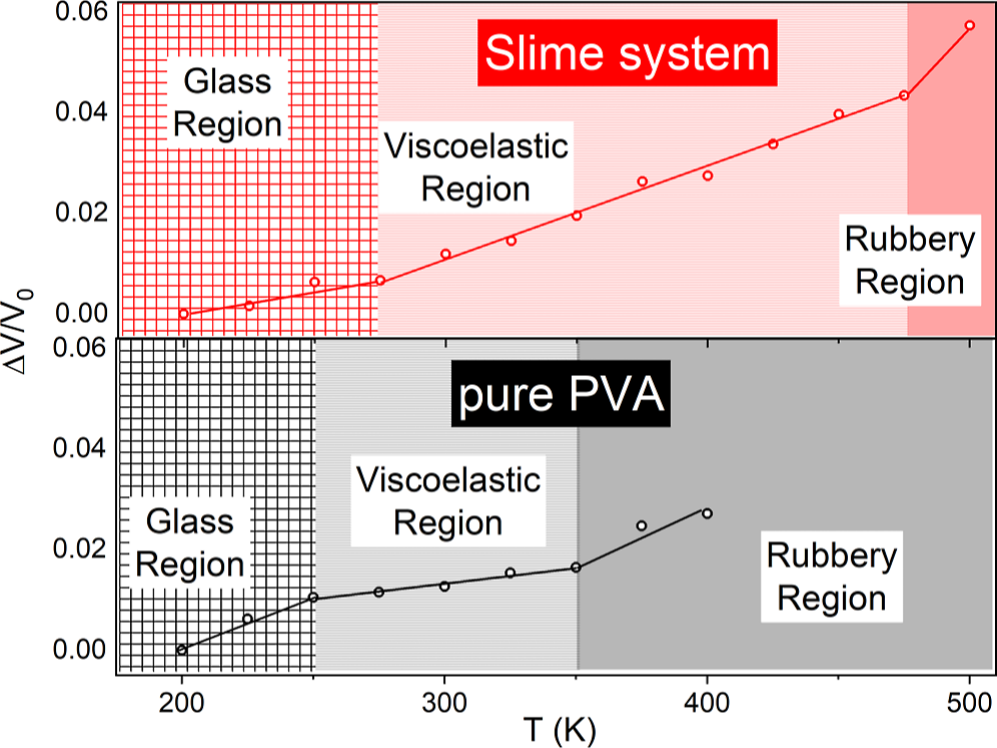
(Color online) Relative volume change at room pressure as a function of temperature, where *V*_0_ refers to volume at 200 K for PVA-pure (bottom) and the Slime system (top).

Our simulations reveal that the viscoelastic region, characteristic of polymers, is wider in the Slime system than in the pure PVA system, which implies that it is an interesting region for barocaloric potential. This wider viscoelastic region suggests a greater fraction of elastic elements within the polymer matrix, contributing to a more gradual and stable mechanical response under pressure. The cross-linking between polymer chains increases their rigidity and reduces the free volume, thereby limiting chain mobility and enhancing the system resistance to pressure-induced deformation. This difference is attributed to the cross-linking between PVA chains, facilitated by tetrahydroxyborate ions causing that transition temperature to be raised.^59^ The radial distribution functions, calculated from the positions of carbon atoms in the intermolecular polymer chains, provide insights into the interactions and proximity between polymer chains in Figure 3 for both pure PVA and Slime systems. The data indicate that in the Slime system, the boron bonds between the chains are in closer proximity than in pure PVA. This closer proximity in the Slime system, as evidenced by a peak at a lower r-value in the RDF, suggests that the boron cross-links promote a tighter packing of PVA monomers, potentially enhancing local density. This structural arrangement contributes to the observed differences in chain interactions of the Slime system compared to pure PVA, reflecting the stabilizing effect of cross-linking on the polymer network. This result is consistent with the idea that pure PVA has a greater free volume between polymer chains, which leads to increased mobility. This decreased molecular mobility in the Slime system, in contrast to pure PVA, is further highlighted by the mean square displacement at 300 K, as depicted in the inset of Figure 3, which is consistent across all temperatures.

**Figure 3:**
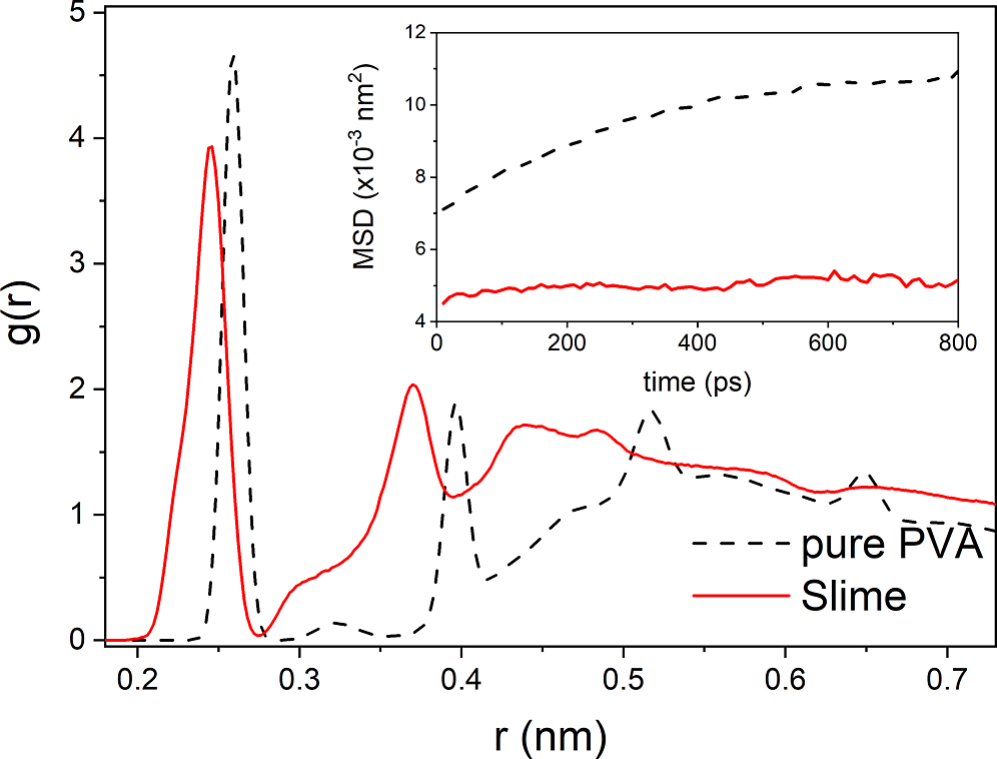
(Color online) Radial distribution function at room pressure for pure PVA (black dashed line) and the Slime system (red solid line). Note that the chains of the Slime system are closer together than the pure PVA chains, and consequently, their mobility decreases, as evidenced by molecule MSD shown in the inset.

To gain deeper insights into the impact of cross-linking on polymer chain proximity and its influence on the barocaloric effect, a comprehensive analysis of the energy contributions in the Slime system will be conducted compared to those in pure PVA. From the molecular perspective, the internal energy of the system is defined as the total energy, denoted by *U* = *E*_total_ = *E*_kinetic_ + *E*_potential_. This includes the kinetic contributions, related to the motion of the polymer chains, and the potential contributions, which encompass both intramolecular (atom within the polymer chains) and intermolecular (atom between different polymer chains) interactions, where the potential energy is given by *E*_potential_ = *E*_intramolecular_ + *E*_intermolecular_. Considering our RDF and MSD results, it is expected that, due to the greater free volume, the kinetic energy contribution of pure PVA is more significant compared to that of the Slime system, primarily because of the higher mobility of the polymer chains. However, the potential energy tends to be higher because of the more substantial intramolecular and intermolecular interactions within and between chains, which can be more significant than the contributions of kinetic energy.

In the left panel of Figure 4, we present the kinetic and potential energies of the pure PVA and Slime systems for 300 K simulations, normalized by the total energy, to demonstrate their contributions more clearly. The results show that the kinetic energy is comparable to the potential energy in pure PVA. In contrast, for the Slime system, the potential energy is considerably higher than the kinetic energy, which is almost negligible, which aligns with our expectations. The closer proximity of polymer chains in the Slime system increases the interactions between them, leading to a significant rise in the potential energy in our calculations. This structural arrangement is consistent with the gel-like state of the Slime system, in which the borate-PVA cross-links were implemented as fixed covalent bonds, preserving the network topology and preventing changes in chain connectivity throughout the simulation. This can be observed in the right panel of Figure 4, where it is shown that the intermolecular energy, including Lennard-Jones and Coulombic interactions, contributes significantly more than the energy from intramolecular interactions. Notably, this energetic behavior is directly influenced by the fixed nature of the Slime cross-links, which constrain large-scale rearrangements and promote closer chain proximity, thereby amplifying intermolecular interactions. Thus, we can conclude that the energy arising from interactions between polymer chains has a more significant impact on the internal energy variations that directly influence the barocaloric effect. Consequently, we can predict that applying pressure, which decreases the distance between the chains, will increase the contribution of the potential energy and its changes.

**Figure 4:**
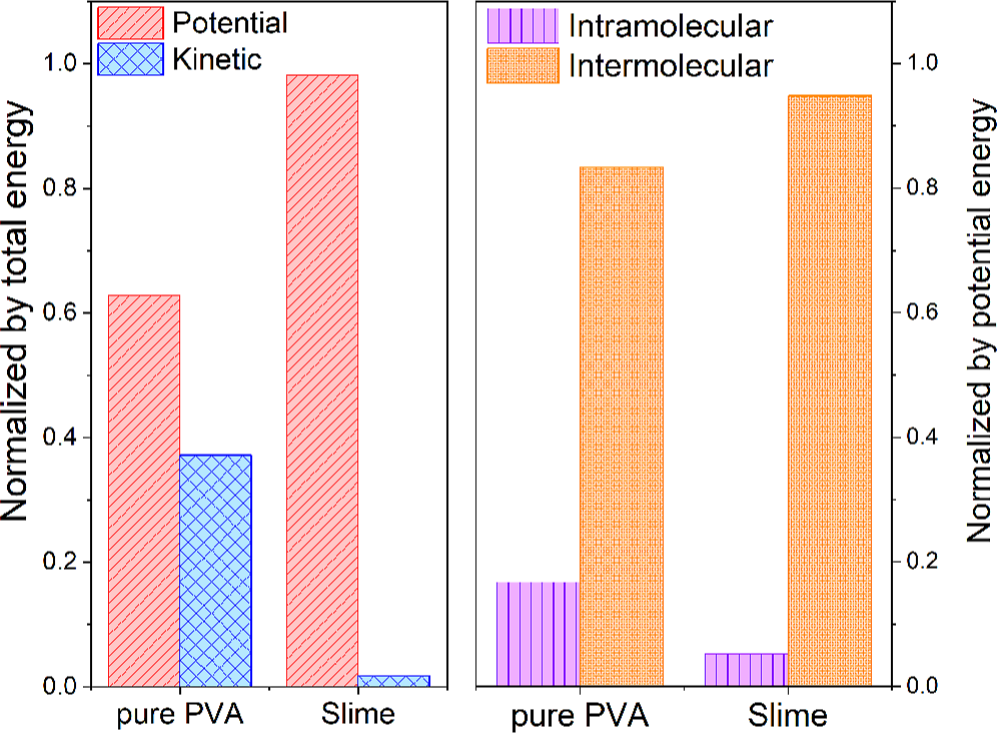
(Color online) Energy found for the simulation obtained under room pressure conditions at 300 K. In the left panel, both kinetic and potential energies normalized by the total energy of the systems are presented. Intramolecular and intermolecular potential energy are shown in the right panel.

Therefore, we perform simulations at various applied pressures to determine the relative volume change for polymer systems, as depicted in Figure 5. For polymer systems, there is an empirical relationship between volume change and applied pressure, known as Tait’s equation, which is given by^60^

7 $$\frac{\Delta V}{V_{0}}=-C\;\ln\left(1+\frac{p}{B}\right)$$

Here, *V*_0_ is the reference volume at zero applied pressure, and *C* (unitless) and *B* (units of pressure) are fitting parameters. The inset in Figure 5 illustrates the adjustment of this relation for both pure PVA and Slime systems at 300 K. The same characteristic is consistent across systems, regardless of the temperature analyzed.

**Figure 5:**
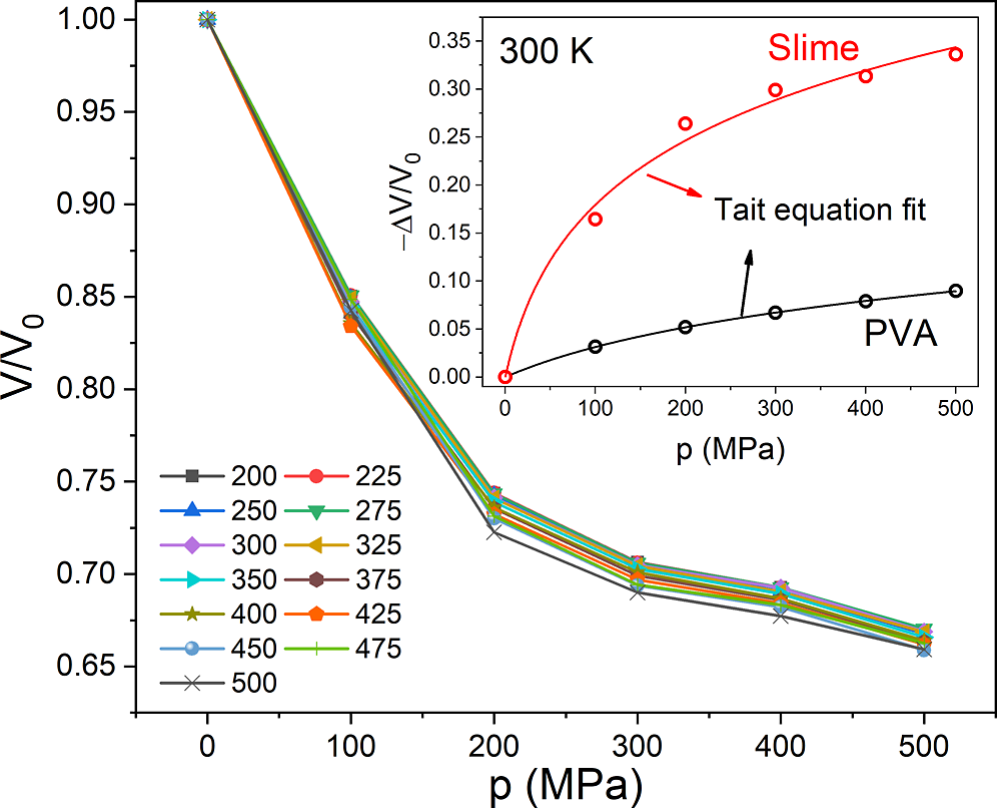
(Color online) Normalized volume, with *V*_0_ taken at 200 K, as a function of simulated applied pressure for the Slime system. The inset shows the relative volume change as a function of pressure at 300 K for pure PVA (black line) and Slime system (red line) adjusted by Tait’s equation, characteristic behavior associated with polymer systems.

From an energetic perspective, we examined how the energy contributions of the systems vary with simulated pressure at a constant temperature, such as 300 K, as illustrated in Figure 6 for pure PVA (bottom) and the Slime system (top). The first significant finding is that the kinetic energy in both systems undergoes negligible changes with increasing pressure. This indicates that the movement of the polymer chains does not affect the energy changes and, consequently, has a limited impact on the barocaloric effect. On the other hand, considering the variations in potential energy, separated into intramolecular and intermolecular components, it is revealed that for pure PVA, the primary contribution to the energy variation comes from the interactions between the polymer chains. This contribution increases with increasing pressure, which is explained by smaller distance chains, such as mentioned above. Similarly, the Slime system also experiences an increase in potential energy with rising simulated pressure, but this increase is more than 10-fold due to smaller distances between polymeric chains. In addition to the elevated intermolecular interactions, there is a notable increase in intramolecular interactions compared to pure PVA, which is attributed to the boron-induced cross-linking between polymer chains.

**Figure 6:**
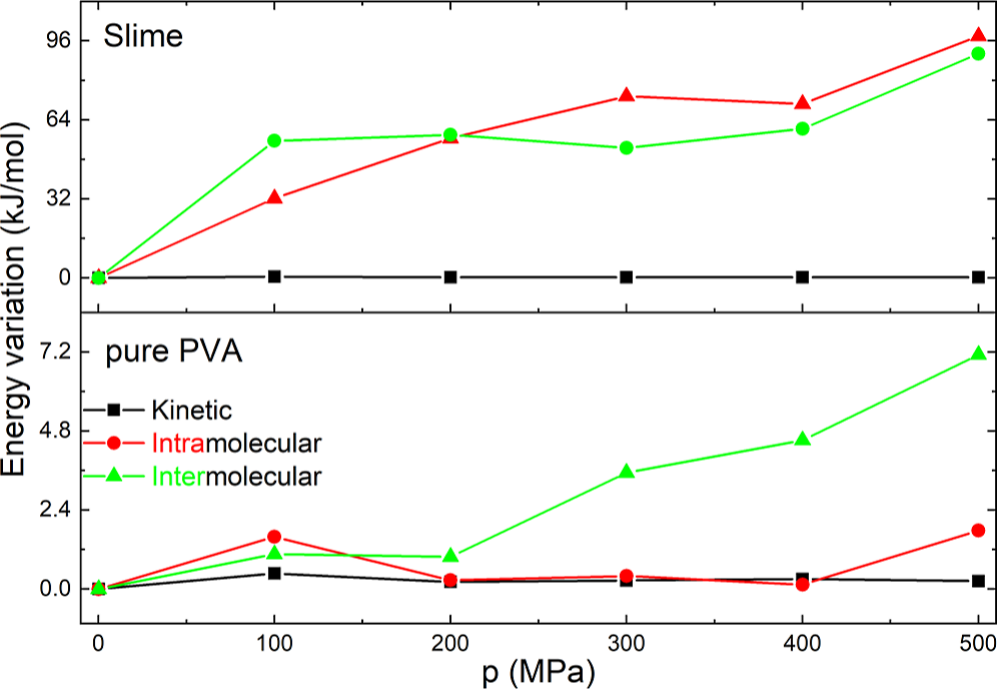
(Color online) Energy variation of kinetic energy and intramolecular and intermolecular potential energy as a function of simulated applied pressure at 300 K for pure PVA (bottom) and the Slime system (top). Take into account that the kinetic energy variation, in both cases, is significantly neglected.

Based on these results, we can anticipate that the barocaloric effect is enhanced in the Slime system due to the increased interaction between polymer chains. This will be further explored in the next section.

### Barocaloric Effect

To investigate the barocaloric effect in polymeric systems, it is essential to consider how applied pressure induces volume changes within the simulation boxes. These volumetric changes lead to thermal responses that give rise to variations in the entropy of the system and corresponding temperature changes, as expressed by eqs 5 and 6. For the analysis of the Slime system, NpT ensembles were computed, with volume changes being monitored as pressure varied from room pressure (≈0.1 MPa) to 500 MPa and temperatures ranged from 200 to 500 K (up to 400 K for pure PVA), resulting in 65 simulation points (45 simulation points for pure PVA). Thus, our findings reveal the behavior of entropy and temperature changes for both pure PVA and Slime systems, in order to investigate the barocaloric effect.

In Figure 7, we present the entropy changes as a function of temperature for various simulated pressures in both pure PVA (left panel) and the Slime system (right panel). It is observed that entropy variation decreases with temperature across all simulated pressure conditions. This result is consistent with our observations above, as the increase in temperature at constant pressure promotes the separation of polymeric chains, thereby reducing intermolecular interactions, which directly affect the energy change and, consequently, the entropy change (Figure 4). Similarly, the increase in pressure strengthens these interactions and, with the temperature kept constant, leads to a greater change in entropy since the polymer chains in the Slime system are more densely closed due to the connections formed by tetrahydroxyborate ions. Consequently, regarding the entropy changes in our Slime system, we observe a more than 4-fold increase compared to that in the pure poly­(vinyl alcohol) system, from 56 JK^–1^ kg^–1^ to 295 JK^–1^ kg^–1^ at the simulated temperature of 300 K and pressure of 300 MPa, which is considered as the giant barocaloric effect. When compared to the other systems investigated, as listed in Table 1, we observe that Li_2_B_12_H_12_ ^23^ and Neopentylglycol (NPG - ${\left({CH}_{3}\right)}_{2}$ C ${\left({CH}_{2}OH\right)}_{2}$ )^21,61^ exhibit a colossal barocaloric effect accompanied by structural changes. On the other hand, in the case of Natural Rubber,^16,22^ it was experimentally found that this behavior is linked to the total internal energy variation without evidence of any phase transition during the process, which is analogously presented in our systems. This characteristic can enhance the efficiency of the barocaloric effect devices since systems without structural transitions tend to exhibit better reversibility, reduced thermal and mechanical hysteresis, and improved stability under repeated pressure cycles. In the context of applications in thermal batteries, entropy changes without associated phase transformation can be an advantage for the design and implementation of more efficient devices, heat storage, and release capacity.

**Figure 7:**
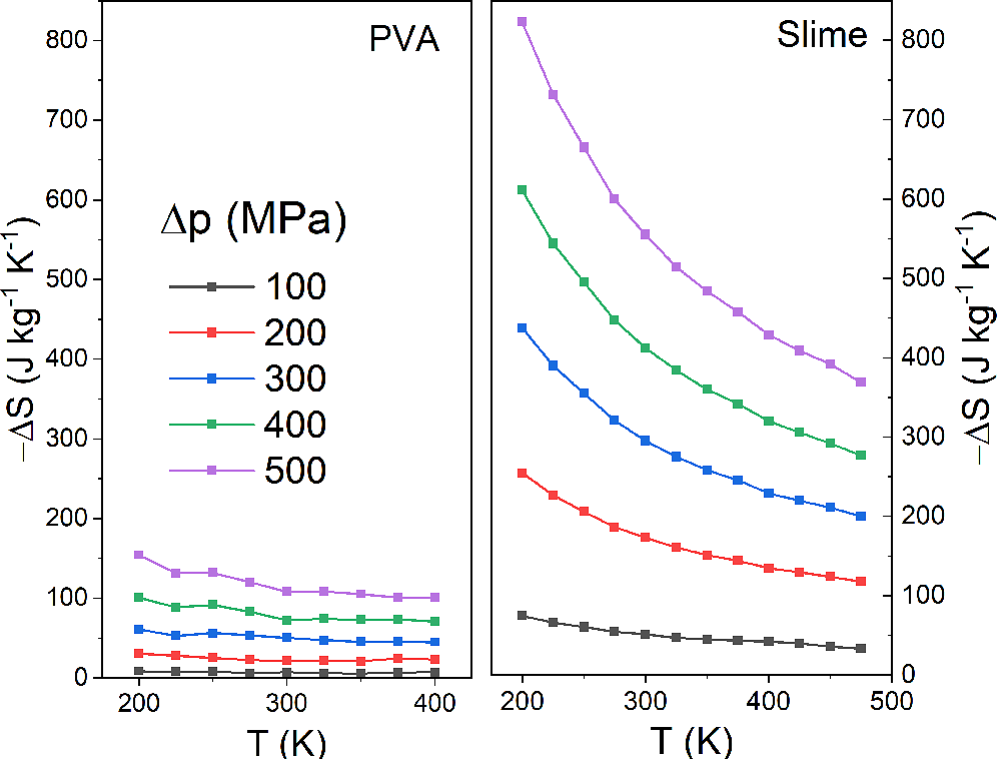
(Color online) Entropy changes as a function of temperature for different variations of simulated applied pressure for pure PVA (left panel) and the Slime system (right panel) at the same scale for better comparison. Consider that the inset highlights the pure PVA data.

**Table 1:** Select Materials with Expressive Barocaloric Effect with Their Respective Entropy Changes and Temperature Variations, Found from Experimental and Molecular Dynamic Simulations, Compared with Our Systems under Similar Conditions

sample	method	*T* (K)	*P* (MPa)	|Δ*S*| (JK^–1^ kg^–1^)	Δ*T* (K)	refs
Li_2_B_12_H_12_	MD simulation	480	100	367	43	^23^
neopentylglycol	experimental	320	250	445	45	^21,61^
$\left({FeL}_{2}\right){\left({BF}_{4}\right)}_{2}$ /PVC	experimental	262	450	168	49	^62^
natural rubber	experimental	300	173	137	25	^16^
natural rubber	MD simulation	545	390	225	25	^22^
pure PVA	MD simulation	300	300	56	3	this work
slime	MD simulation	300	300	295	26	this work

Another quantity that helps us understand our systems’ barocaloric effect is the temperature adiabatic changes from an initial temperature when external pressure is applied.^63^ Interestingly, for our calculation of pure PVA and Slime systems, the change in temperature due to the barocaloric effect remains almost constant regardless of the absolute temperature of the system when the pressure is constant. This is possible since the adiabatic bulk modulus and thermal expansion do not vary with temperature when the applied pressure is constant. This effect is determined solely by the change in pressure, as is illustrated in Figure 8. Thus, our results reveal that the temperature change is intrinsically linked to the applied pressure, which suggests that the relative impact of the pressure change on the temperature is uniform throughout the range of temperatures considered. Once again, the Slime system exhibits a more pronounced temperature variation; for instance, at 300 K and 300 MPa, the maximum temperature change was 26 K, notably greater than the 3 K variation observed in pure PVA. These findings have been observed experimentally in materials such as NPG and $\left({FeL}_{2}\right){\left({BF}_{4}\right)}_{2}$ /PVC compounds,^62^ which are known, respectively, as a plastic crystal and a spin crossover polymer composite. In the same way, molecular dynamics simulations were used in Li_2_B_12_H_12_ to obtain analogous results. To further investigate the effect of pressure on temperature change, we analyzed the relationship between these two quantities, which revealed a linear relationship with the form Δ*T* = (0.0110 MPa^–1^ p - 6.5) K, as illustrated in the video in Supporting Information (S2). These results are useful for practical applications such as solid-state refrigeration systems, as they allow the temperature change of the material to be accurately predicted under hydrostatic mechanical pressure, regardless of the initial temperature. This prediction can also be extended to other cross-linked polymeric systems beyond PVA and slime.

**Figure 8:**
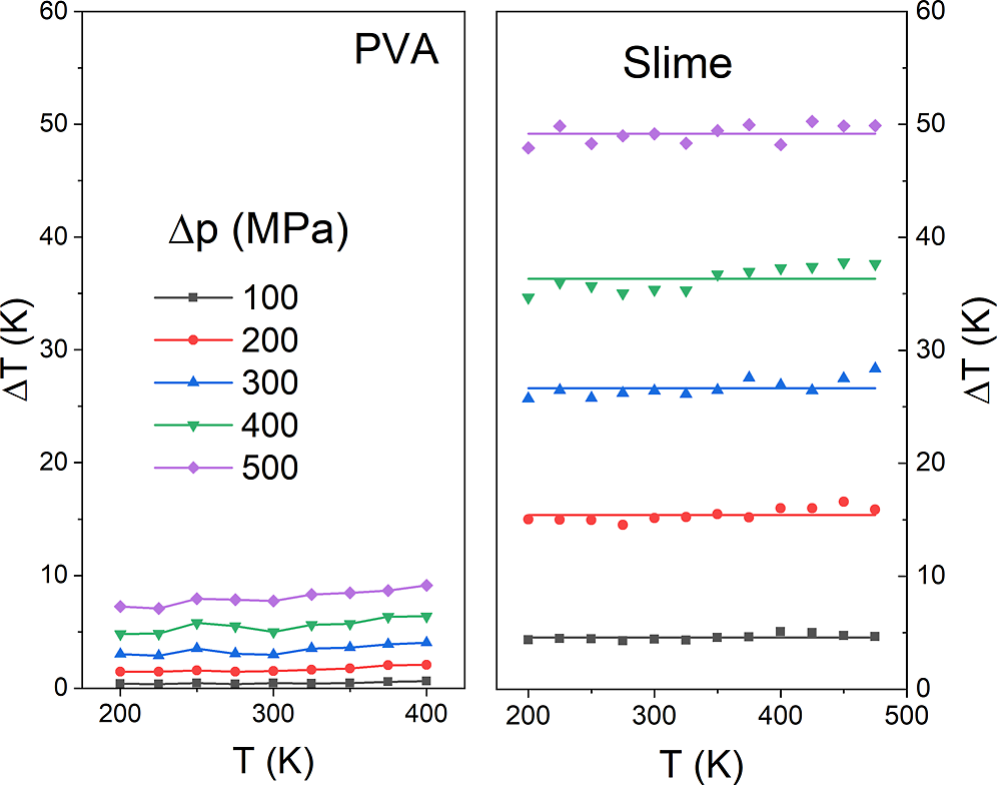
(Color online) Temperature changes, when applied pressure, as a function of temperature for pure PVA (left panel) and the Slime system (right panel). The left inset refers to the pure PVA data, while the right inset shows the behavior of temperature changes as a function of pressure variation adjusted by a linear fit (solid black line).

## Conclusions

In this work, we developed polymeric models based on pure PVA and PVA/Borax polymer chains, resulting in a Slime system. The analysis of these systems provided important insights into how volume variations respond to changes in the pressure and temperature. Our characterizations revealed that structural properties, such as radial distribution and atomic mobility, are significantly influenced by the cross-links between the polymer chains in the investigated systems. These bonds are mostly facilitated by tetrahydroxyborate ions present in the Slime system, reducing the mobility of the polymer chains, and evidenced by changes in the mean square displacement and the proximity between chains, promoting greater intermolecular interaction between them. Based on our energetic analysis, we found that the motion of the polymer chains does not significantly affect the energy changes, limiting their impact on the barocaloric effect; on the other hand, the variations in potential energy reveal that the proximity between chains produces a greater variation in the total internal energy of the systems and, consequently, due to the bonds with the boron atoms for the Slime system, a greater contribution to the barocaloric effect.

The analysis of the barocaloric effect in the pure PVA and Slime systems reveals significant results, in terms of both entropy changes and temperature variations under pressure variations. Our results show that the entropy variation in these systems decreases with increasing temperature, which is consistent with the separation of the polymer chains and the decrease of intermolecular interactions at higher temperatures. On the other hand, increasing the pressure variation generates higher values of the entropy changes. In particular, the Slime system exhibits a remarkably larger barocaloric effect than the pure PVA, with a more than 4-fold increase in entropy at 300 K and 300 MPa, indicating a direct effect of the proximity of the polymer chains in this barocaloric system. Furthermore, the temperature variation induced by the barocaloric effect shows a direct dependence on the applied pressure, without depending on the initial temperature of the system. This finding, evidenced by the linear behavior between the pressure and temperature changes, suggests that the barocaloric effect in the investigated systems can be accurately predicted in practical applications such as solid-state cooling systems. These behaviors, similar to those observed in natural rubber,^16,62^ are evidenced without any phase transition during the process, which introduces new possibilities to the development of materials for barocaloric effect applications. Thus, these findings aim to motivate future experimental studies on cross-linking influence to optimize the barocaloric response of polymer-based materials.

## Supplementary Material





## Notes

Additional data that complement the findings presented in the manuscript, offering further insights and supporting analyses, including the force field parameters employed in the simulations, along with example files used to set up both the PVA and Slime systems (ZIP)
Video illustrating the linear temperature change as a function of applied pressure variations for the Slime system (MP4)
