# Supplementary material for: Evidence of the Giant Barocaloric Effect in the PVA-Slime System by Molecular Dynamics Simulations
Source: ACS Omega. 2025 Aug 23;10(35):39580–8. doi: 10.1021/acsomega.5c02475 (PMC12423886; doi:10.1021/acsomega.5c02475)
Supplement: Supplementary file 1 [file ao5c02475_si_001.zip › Fig S1.docx]

**Evidence of giant barocaloric effect in PVA-Slime system by molecular dynamics simulations**

Richard J. Caraballo-Vivas,∗,† Marcelo Albuquerque,∗,† Vanessa Torres,∗,† Luciano T. Costa,∗,‡ Pedro Venezuela,∗,† and Mario S. Reis∗,†

*†Institute of Physics, Universidade Federal Fluminense, Av. Gal. Milton Tavares de Souzas/n, 24210-346, Niter´oi-RJ, Brazil*

*‡MolMod-CS–Institute of Chemistry, Universidade Federal Fluminense, Outeiro de S˜ao Jo˜ao Batista, CEP: 24020-141, Niter´oi RJ, Brazil*

E-mail: caraballorichard@gmail.com; marcelofilho@id.uff.br; vanessa185@gmail.com; ltcosta@id.uff.br; pedrovenezuela@id.uff.br; marioreis@id.uff.br


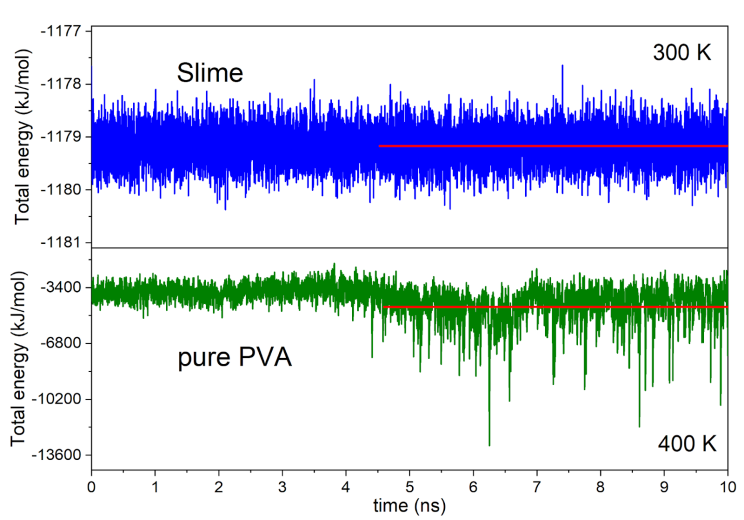


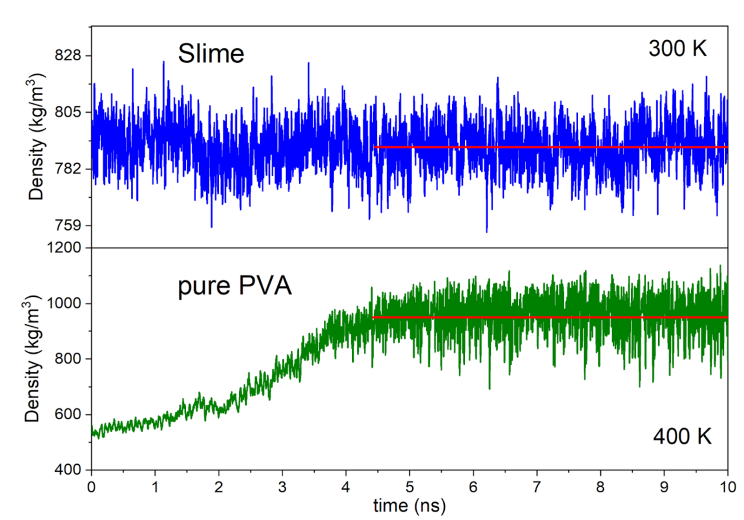


Fig. S1. Equilibration stage of the simulated systems under NpT ensemble using a C-rescale barostat, at room pressure and 300 K for Slime system and 400 K for pure PVA. The bottom panel shows the density evolution over time, while the top panel presents the fluctuation of the total energy.
